# Incorporating Genetic Heterogeneity in Whole-Genome Regressions Using Interactions

**DOI:** 10.1007/s13253-015-0222-5

**Published:** 2015-11-09

**Authors:** Gustavo de los Campos, Yogasudha Veturi, Ana I. Vazquez, Christina Lehermeier, Paulino Pérez-Rodríguez

**Affiliations:** Department of Epidemiology & Biostatistics, Michigan State University, 909 Fee Road, Room B601, East Lansing, MI 48824 USA; Department of Statistics & Probability, Michigan State University, 619 Red Cedar Rd., East Lansing, MI 48824 USA; University of Alabama at Birmingham, Ryals Public Health Bldg. 443, Birmingham, AL 35294 USA; Department of Plant Breeding, Technische Universität München, Liesel-Beckmann-Str. 2, 85354 Freising, Germany; Colegio de Postgraduados, Km. 36.5, Carretera Mexico, Montecillo, 56230 Texcoco, Estado de México Mexico

**Keywords:** Genomic prediction, Genomic selection, Population structure, Multi-breed analysis, Bayesian

## Abstract

Naturally and artificially selected populations usually exhibit some degree of stratification. In Genome-Wide Association Studies and in Whole-Genome Regressions (WGR) analyses, population stratification has been either ignored or dealt with as a potential confounder. However, systematic differences in allele frequency and in patterns of linkage disequilibrium can induce sub-population-specific effects. From this perspective, structure acts as an effect modifier rather than as a confounder. In this article, we extend WGR models commonly used in plant and animal breeding to allow for sub-population-specific effects. This is achieved by decomposing marker effects into main effects and interaction components that describe group-specific deviations. The model can be used both with variable selection and shrinkage methods and can be implemented using existing software for genomic selection. Using a wheat and a pig breeding data set, we compare parameter estimates and the prediction accuracy of the interaction WGR model with WGR analysis ignoring population stratification (across-group analysis) and with a stratified (i.e., within-sub-population) WGR analysis. The interaction model renders trait-specific estimates of the average correlation of effects between sub-populations; we find that such correlation not only depends on the extent of genetic differentiation in allele frequencies between groups but also varies among traits. The evaluation of prediction accuracy shows a modest superiority of the interaction model relative to the other two approaches. This superiority is the result of better stability in performance of the interaction models across data sets and traits; indeed, in almost all cases, the interaction model was either the best performing model or it performed close to the best performing model.

**Electronic Supplementary Material:**

Supplementary materials for this article are available at 10.1007/s13253-015-0222-5.

## Introduction

Genomic selection (GS) (Meuwissen et al. 2001) has been adopted in animal and plant breeding at a relatively fast pace (e.g., de los Campos et al. 2013). The classical quantitative genetics model (e.g., Falconer and Mackay 1996; Lynch and Walsh 1998) and the statistical methods commonly used for GS are defined with reference to a homogeneous population. For instance, in a standard GS model, the regression of phenotypes, $\varvec{y}=\left\{ {y_i } \right\} \left( {i=1,\ldots ,n} \right)$, on markers, $\varvec{X}=\left\{ {x_{ij} } \right\} \left( {j=1,\ldots ,p} \right)$, is assumed to be homogeneous across subjects in the sample, that is, $\varvec{y}=\varvec{X}\varvec{\beta } +\varvec{\varepsilon }$, where $\varvec{\beta } =\left\{ {\beta _j } \right\}$ and $\varepsilon =\left\{ {\varepsilon _i } \right\}$ are vectors of marker effects and model errors, respectively. However, many plant and animal breeding populations exhibit various types and levels of structure.

In Genome-Wide Association Studies (GWAS), population structure has been viewed as a potential confounder (e.g., Astle and Balding 2009). To mitigate the inferential problems that population structure can bring, multiple methods have been proposed, including principal components regressions as well as mixed-model analyses, where population structure and substructure are accounted for by introducing random effects (e.g., Price et al. 2010). Both approaches assume homogeneity of effects across sub-populations; however, differences in allele frequencies and in linkage-disequilibrium patterns may make both QTL and marker effects vary between sub-populations (de los Campos and Sorensen 2014). From this perspective, structure acts as an effect ‘modifier’ rather than as a confounder.

Genetic heterogeneity can be addressed using a stratified analysis; however, such an approach is limited by the strata sample size, and it is well established that sample size is one of the most important factors limiting prediction accuracy in GS. Moreover, a stratified analysis does not shed light on the degree of genetic similarity/differentiation between sub-populations.

Recently, there has been an increased interest in the combined analysis of data from several sub-populations (e.g., multi-breed and multi-country data). Hayes et al. (2009)’s study was one of the first studies that considered analyzing data from multiple populations jointly. In that study, the authors compared the prediction accuracy of a multi-breed analysis with a stratified (i.e., within-breed) analysis using data from Holsteins and Jerseys. The results of Hayes et al. (2009) suggested no benefits of the combined analysis on prediction accuracy for the breed with larger reference panel (Holstein) and a small gain in prediction accuracy for the group with smaller reference panel (Jersey). However, the multi-breed analysis presented in Hayes et al. (2009), as well as in other studies such as that of Daetwyler et al. (2010), was based on the standard GS models where the effects of allele substitutions were assumed to be a constant across breeds. This assumption may have offset the benefits of having a larger reference panel, thus, limiting the potential benefits of conducting a multi-breed analysis.

The recognition that effects may vary between groups has led to a second wave of studies based on multivariate models where effects are allowed to be different, but correlated, across groups (e.g., Olson et al. 2012; Karoui et al. 2012). This has been done exclusively within a Gaussian context, in part because dealing with multivariate models for high-density marker panels outside of the “Gaussian world” is not straightforward.

In this study we propose to deal with genetic heterogeneity using a marker-by-cluster random effects interaction model. Relative to standard multivariate Gaussian models such as those already applied to the analysis of multi-breed data, the interaction model has several appealing features. Firstly, it can be applied with both shrinkage and variable selection methods. Secondly, it decomposes marker effects into components that are constant across groups and deviations that are group-specific. Therefore, in principle, the proposed approach could be used to identify regions that have constant effects across groups and ones that exhibit substantial interaction.

The rest of this article is organized as follows: Sect. 2 presents a motivating example based on wheat data. In Sect. 3, we describe the proposed interaction model. In Sect. 4, we introduce the two data sets that were used to evaluate the model and describe the analyses that were carried out. Results are presented in Sect. 5. Finally, a discussion of the results and a few perspectives on the advantages and limitations of the proposed model are offered in Sect. 6.

## Motivating Example

**Fig. 1:**
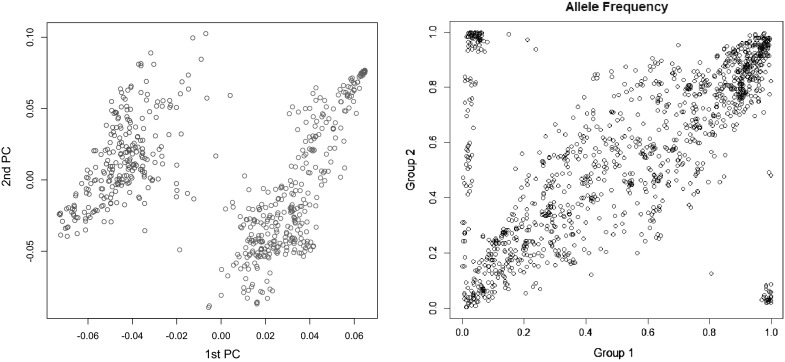
Clustering in the Wheat data set. First two marker-derived principal components (*left*) and allele frequency by group (*right*).

To motivate the problem, we present an example based on a wheat (*Triticum*) data set from CIMMYT comprising 599 pure lines of wheat that was genotyped for 1279 DArT markers (Triticarte Pty. Ltd., Canberra, Australia http://www.triticarte.com.au). This data set has been used for GS data analyses multiple times (e.g., de los Campos et al. 2009; Crossa et al. 2010). The data set presents a strong structure (Janss et al. 2012). Figure 1 (right panel) displays the first two marker-derived principal components; the two colors correspond to clusters derived from the markers using the R-package PSMix (Wu et al. 2006). The first two eigenvalues explained 16.3 % of the sum of the eigenvalues, and the first two eigenvectors clearly cluster lines into two distinctive groups. The right panel of Fig. 1 shows allele frequencies by cluster; a large proportion of the markers have markedly different allele frequencies between groups; and 22 % of the markers had observed allele frequencies in groups 1 and 2 that differ by more than 0.2 units. Loosely speaking, there are three groups of markers: the great majority of the loci have observed allele frequencies that cluster in a cloud along the 45-degree line, this cloud has a rhomboid shape, with maximum variance at intermediate allele frequencies and smaller variance at extreme allele frequencies. This is the pattern expected under no-differentiation: allele frequencies in each sample vary due to sampling variance, and the sampling variance is maximum at intermediate allele frequencies. A second group of markers appearing in the lower right and upper left corners of the right plot of Fig. 1 are fixed or almost fixed for opposite alleles at groups 1 and 2. Finally, a third group of markers (appearing along a vertical line near the abscissa of zero) are almost fixed in group 1 and have allele frequencies ranging from 0.2 to 0.8 in group 2. The last two groups of markers are clearly differentiated between populations. When dominance and epistasis are present, the regression of genetic values on allele content (or average effect of allele substitution) is allele frequency dependent (the reader is referred to Lynch and Walsh 1998, p. 68 for further details). Consequently, due to dominance and epistasis, both groups are likely to have different effects of allele substitution at those loci, if such effects are not explicitly modeled.

**Fig. 2:**
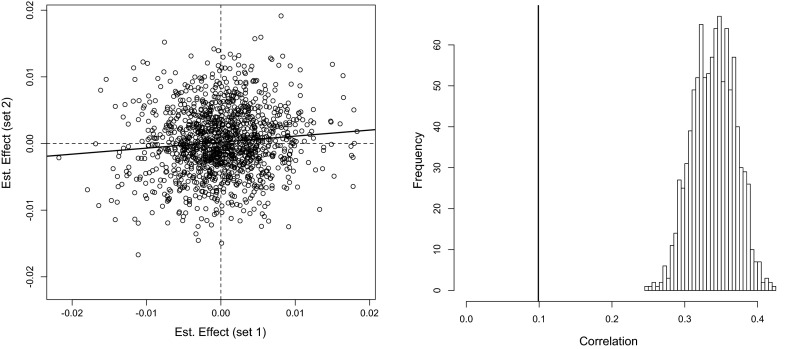
Scatter plot of estimated effects obtained with a stratified analysis (*left*) and estimated sampling distribution of the correlation between estimated effects obtained with 1000 permutations (*right*).

### Stratified Analysis

Given the important level of stratification present in this data set, it is reasonable to expect that QTL and marker effects may vary between sub-populations. To assess this hypothesis, we estimated marker effects using ridge regression within cluster; the results from this analysis are reported in the left panel of Fig. 2. The sample correlation of the estimated effects was only 0.1, suggesting that marker effects are markedly different between clusters. However, estimated effects are subject to sampling errors; consequently, the absolute value of the sample correlation between estimated effects under-estimates the absolute value of the correlation between the (unknown) true effects. Therefore, we cannot rule out the possibility that the low correlation observed between estimated effects only reflects the low precision of estimates. To assess whether or not this is the case, we conducted 1000 permutations by re-shuffling the cluster labels. This exercise yielded 1000 samples of the correlation between estimates obtained in a scenario where the group label has no relationship with the genetic architecture. A histogram of the 1000 correlations obtained is displayed in the right panel of Fig. 2. The average and median correlations from the permutation distribution were both 0.34, and the 2nd percentile was 0.28. We conclude that the correlation of estimates obtained when the groups were derived from the observed genotypes does not belong to the distribution obtained with the permutation. This suggests that indeed, as one may suspect from the inspection of principal components and the distribution of allele frequencies, marker effects vary between clusters.

## Statistical Model

In this section, we describe an extension of the standard whole-genome regression model (Meuwissen et al. 2001) that accommodates genetic heterogeneity by decomposing marker effects into terms that are common between clusters and random deviations that are cluster-specific. For ease of presentation, we assume that subjects can be clustered into two groups and that the only effects to be accounted for are the cluster means and marker effects; extensions to more than two groups and to models that accommodate other sources of variation (e.g., effects associated with experimental design factors or other systematic effects) are relatively straightforward.

### Interaction Model

In this model, we assume that marker effects have two components: one that is common across clusters ($$b_{0j} $$, where $$j=1,{\ldots },p$$ indexes markers) and one that is cluster-specific ($$b_{1j} $$ and $$b_{2j} $$ for groups 1 and 2), respectively. Therefore, marker effects become $$\beta _{1j} =b_{0j} +b_{1j} $$ and $$\beta _{2j} =b_{0j} +b_{2j} $$ in groups 1 and 2, respectively. With these, the data equations for groups 1 and 2 become

1 $$\begin{aligned} \left[ {{\begin{array}{l} {\varvec{y}_1 } \\ {\varvec{y}_2 } \\ \end{array} }} \right] =\left[ {{\begin{array}{l} {\varvec{1}\mu _1 } \\ {\varvec{1}\mu _2 } \\ \end{array} }} \right] +\left[ {{\begin{array}{l} {\varvec{X}_1 } \\ {\varvec{X}_2 } \\ \end{array} }} \right] \varvec{b}_0 +\left[ {{\begin{array}{l} {\varvec{X}_1 } \\ \varvec{0} \\ \end{array} }} \right] \varvec{b}_1 +\left[ {{\begin{array}{l} \varvec{0} \\ {\varvec{X}_2 } \\ \end{array} }} \right] \varvec{b}_2 +\left[ {{\begin{array}{l} {\varvec{\varepsilon } _1 } \\ {\varvec{\varepsilon } _2 } \\ \end{array} }} \right] , \end{aligned}$$

where $\varvec{y}_1 =\left\{ {y_{1i} } \right\} _{i=1}^{n_1 }$, $\varvec{y}_2 =\left\{ {y_{2i} } \right\} _{i=1}^{n_2 }$, $\varvec{X}_1 =\left\{ {x_{1ij} } \right\} _{i=1,j=1}^{n_1 ,p}$, and $\varvec{X}_2 =\left\{ {x_{2ij} } \right\} _{i=1,j=1}^{n_2 ,p}$ represent the phenotypes and genotypes of individuals in groups 1 and 2, respectively; $\mu _1$ and $\mu _2$ represent cluster-specific intercepts; $\varvec{b}_0 =\left\{ {b_{0j} } \right\}$, $\varvec{b}_1 =\left\{ {b_{1j} } \right\}$, and $\varvec{b}_2 =\left\{ {b_{2j} } \right\}$ are vectors of marker effects and $\varepsilon _1 =\left\{ {\varepsilon _{1i} } \right\}$; and $\varepsilon _2 =\left\{ {\varepsilon _{2i} } \right\}$ represent model errors.

### Likelihood

The error terms are assumed to be independently and normally distributed with zero mean and cluster-specific variances ($\sigma _1^2$ and $\sigma _2^2 )$; therefore, the likelihood function becomes

2 $$\begin{aligned} p\left( {\varvec{y}_1 ,\varvec{y}_2 |\mu _1 ,\mu _2 ,\varvec{b}_0 ,\varvec{b}_1 ,\varvec{b}_2 ,\sigma _1^2 ,\sigma _2^2 } \right)= & {} {\prod }_{i=1}^{n_1 } N\left\{ {y_{1i} |{\sum }_{j=1}^p x_{1ij} \left( {b_{0j} +b_{1j} } \right) ,\sigma _1^2 } \right\} \nonumber \\&\times \, {\prod }_{i=1}^{n_2 } N\left\{ {y_{2i} |{\sum }_{j=1}^p x_{2ij} \left( {b_{0j} +b_{2j} } \right) ,\sigma _2^2 } \right\} .\nonumber \\ \end{aligned}$$

### Prior

In whole-genome regression models, marker effects are usually assigned IID priors (de los Campos et al. 2013); here we follow this approach but assign distinct priors to the main and interaction effects. For other unknowns (intercepts and error variances), we adopt standard conjugate priors. The joint prior distribution is assumed to be as follows:

3 $$\begin{aligned}&p\left( {\varvec{b}_0 ,\varvec{b}_1 ,\varvec{b}_2 ,\mu _1 ,\mu _2 ,\varOmega _0 ,\varOmega _1 ,\varOmega _2 ,\sigma _1^2 ,\sigma _2^2 } \right) \nonumber \\&\quad \propto {\prod }_{j=1}^\mathrm{p} p\left( {b_{0j} |\varOmega _0 } \right) p\left( {b_{1j} |\varOmega _1 } \right) p\left( {b_{2j} |\varOmega _2 } \right) \nonumber \\&\qquad \times p\left( {\varOmega _0 } \right) p\left( {\varOmega _1 } \right) p\left( {\varOmega _2 } \right) \chi ^{-2}\left( {\sigma _1^2 |\hbox {S}_1 ,\hbox {df}_1 } \right) \chi ^{-2}\left( {\sigma _2^2 |\hbox {S}_2 ,\hbox {df}_2 } \right) , \end{aligned}$$

where $p\left( {b_{0j} |\varOmega _0 } \right)$, $p\left( {b_{1j} |\varOmega _1 } \right)$, and $p\left( {b_{2j} |\varOmega _2 } \right)$ are the prior densities assigned to the main effects and interaction terms applying to groups 1 and 2, respectively. These can be any of the priors commonly used in GS models, including the Gaussian (G-BLUP or BRR= “Bayesian Ridge Regression,” e.g., VanRaden 2008), double exponential (Bayesian Lasso, Park and Casella 2008), scaled-t (BayesA, Meuwissen et al. 2001), and finite mixture with a point of mass at zero and a slab that can either be a Gaussian (BayesC, Habier et al. 2011) or a scaled-t (BayesB, Meuwissen et al. 2001) density. Importantly, in (3), the main effects and interaction terms are assigned different priors, each of which is indexed by its own regularization parameters, $\varOmega _.$. The parameters included in $\varOmega _.$ depend on the prior assigned to the effects. For instance, if the prior is Gaussian, $\varOmega _.$ is a variance; in BayesA, $\varOmega _.$ includes the degree of freedom and scale parameters of the t-distribution, and in finite mixture models, $\varOmega _.$ includes both the hyper parameters indexing the slab and the prior proportion of non-null effects. In all cases, $\varOmega _.$ can have strong influences on inferences; therefore, rather than specifying them *a priori*, we treat these as random and infer them from the data. Finally, $\chi ^{-2}\left( {\sigma _k^2 |S_k ,df_k } \right) \left( {k=1,2} \right)$ denotes a scaled-inverse Chi-square prior assigned to the $k^\mathrm{th}$ error variance with scale parameter $S_k >0$ and degree of freedom parameter $df_k >0$.

### Posterior Density

With the assumptions described (expression (1)–(3)), the posterior density of the model becomes:

4 $$\begin{aligned}&p\left( {\varvec{b}_0 ,\varvec{b}_1 ,\varvec{b}_2 ,\sigma _1^2 ,\sigma _2^2 |\varvec{y}_1 ,\varvec{y}_2 ,\varvec{d}_1,\varvec{d}_2 } \right) \nonumber \\&\propto \mathop \prod \limits _{i=1}^{n_1 } N\left\{ {y_{1i} \hbox {|}\mathop \sum \limits _{j=1}^p x_{1ij} \left( {b_{0j} +b_{1j} } \right) ,\sigma _1^2 } \right\} \mathop \prod \limits _{i=1}^{n_2 } N\left\{ {y_{2i} \hbox {|}\mathop \sum \limits _{j=1}^p x_{2ij} \left( {b_{0j} +b_{2j} } \right) ,\sigma _2^2 } \right\} \nonumber \\&\qquad \times \left\{ {\mathop \prod \limits _j^\mathrm{p} p\left( {b_{0j} |\varOmega _0 } \right) p\left( {b_{1j} |\varOmega _1 } \right) p\left( {b_{2j} |\varOmega _2 } \right) } \right\} \nonumber \\&\qquad \times p\left( {\varOmega _0 } \right) p\left( {\varOmega _1 } \right) p\left( {\varOmega _2 } \right) \chi ^{-2}\left( {\sigma _1^2 |\hbox {S}_1 ,\hbox {df}_1 } \right) \chi ^{-2}\left( {\sigma _2^2 |\hbox {S}_2 ,\hbox {df}_2 } \right) . \end{aligned}$$

This posterior distribution does not have a closed form; however, samples can be generated using a Gibbs sampler. The current version of the BGLR package (Pérez-Rodriguez and de los Campos 2014) can be used to implement this model; importantly, the package implements a wide range of priors including the G-BLUP, BayesA–B and –C models. Version 1.03 of BGLR did not implement heterogeneous error variance models. For this article, we have developed a new version of BGLR (v1.04) that fits heterogeneous error variance models, as needed to implement the model in expression (4).

### Across-Group Analysis (A)

A standard GS model for an across-group analysis can be obtained by setting $\varvec{b}_1 =\varvec{b}_2 =\varvec{0}$; therefore, the data equations become

5 $$\begin{aligned} \left[ {{\begin{array}{l} {\varvec{y}_1 } \\ {\varvec{y}_2 } \\ \end{array} }} \right] =\left[ {{\begin{array}{l} {\varvec{1}\mu _1 } \\ {\varvec{1}\mu _2 } \\ \end{array} }} \right] +\left[ {{\begin{array}{l} {\varvec{X}_1 } \\ {\varvec{X}_2 } \\ \end{array} }} \right] \varvec{b}_0 +\left[ {{\begin{array}{l} {\varvec{\varepsilon }_1 } \\ {\varvec{\varepsilon }_2 } \\ \end{array} }} \right] \end{aligned}$$

### Stratified Analysis

A within-group analysis can be obtained by assuming $\varvec{b}_0 =\varvec{0}$; in this case, the data equations become

6 $$\begin{aligned} \left[ {{\begin{array}{l} {\varvec{y}_1 } \\ {\varvec{y}_2 } \\ \end{array} }} \right] =\left[ {{\begin{array}{l} {\varvec{1}\mu _1 } \\ {\varvec{1}\mu _2 } \\ \end{array} }} \right] +\left[ {{\begin{array}{l} {\varvec{X}_1 } \\ \varvec{0} \\ \end{array} }} \right] \varvec{b}_1 +\left[ {{\begin{array}{l} \varvec{0} \\ {\varvec{X}_2 } \\ \end{array} }} \right] b_2 +\left[ {{\begin{array}{l} {\varvec{\varepsilon }_1 } \\ {\varvec{\varepsilon }_2 } \\ \end{array} }} \right] . \end{aligned}$$

## Data Analyses

We applied the models defined by expressions (1), (5), and (6) to wheat and pig (*Sus crofa*) breeding data; each of these data sets is briefly described next.

### Wheat Data

This data set is the one used in the motivating example. Briefly, the wheat data set comprises genotypes ($p=1279$ markers) and phenotypes for $n=599$ wheat lines from CIMMYT’s international breeding program. Phenotypes consist of grain yield evaluated in four different environments. We analyzed data from each of those environments separately. Further details about this data set can be found in Crossa et al. (2010).

### Pig Data

This data set was generated by the Pig Improvement Company (http://www.pic.com, a Genus company), and it was made publicly available by Cleveland et al. (2012). These data provide genotypes ($p=50{,}436$) for $n=3534$ individuals. Here, we analyzed three phenotypes labeled by Cleveland et al. as T3, T4, and T5.

### Data Analyses

In both cases, before any analyses were done, genotypes were centered to a null sample mean and scaled to a unit sample variance (this was done by subtracting from the original genotypes the sample mean of each marker and dividing the resulting centered genotypes by the sample standard deviation of the marker). Centering and standardization were done across group (i.e., using the average genotype and its standard deviation in the entire sample).

Our analyses included three main steps: (i) Clustering of materials based on the available genotypes, (ii) parameter estimation using all the available data for each data set, and (iii) assessment of prediction accuracy based on replicated training-testing evaluations.

(i) *Clustering* First, for each data set, we computed a genomic relationship (***G***) based on centered and scaled marker genotypes, and from this matrix, we extracted the first two principal components (PCs). Genomic relationships were computed as $\varvec{G}=p^{-1}\varvec{WW}'$, where $\varvec{W}$ is a matrix containing scaled and centered genotypes; centering and scaling were performed based on the sample mean and sample SD computed using genotypes from all the sub-populations included in the study. In both data sets, the first two PCs show clear evidence of structure: two groups in the wheat data set and three groups in the pig data set. Subsequently, we clustered genotypes using the R-package PSMix (Wu et al. 2006). In the case of the wheat data set, the clustering was based on all the available markers. In the pig data set, the number of markers is much larger; therefore, we first selected a subset of 674 weakly correlated markers (only the set of markers that had pair wise correlations smaller than 0.1), and these were used to cluster genotypes.

(ii) *Estimation* The models of expressions (1), (5), and (6) were fitted to each of the data sets using BRR and BayesB. In both cases, we used the default settings of BGLR (Pérez-Rodriguez and de los Campos 2014); specifically, variance parameters were treated as random, and in model BayesB, the degree of freedom parameter was fixed at a value of 5, and the scale and the proportion of non-null effects were treated as random. First, models were fitted to the entire data set; from these analyses we report estimates of the error variance and of the parameters indexing the prior distributions of effects. As stated, in all analyses, markers genotypes were centered by subtracting the sample mean, and standardized by dividing genotypes by the square root of the sum of the sample variance of the marker genotypes. With this standardization, the variance parameters involved in the Gaussian model can be interpreted as ‘genomic variances.’ In the wheat data set, two clusters were identified; therefore, the interaction model was as described in Eq. (1). On the other hand, in the pig data set, three clusters were identified; therefore, in this case, the interaction model of Eq. (1) was extended by adding a third interaction term for the third cluster.

(iii) *Assessment of Prediction Accuracy* Subsequently, the models of expressions (1), (5), and (6) were fitted on training data sets (TRN), and prediction accuracy was assessed in testing data sets (TST). We conducted 50 TRN-TST experiments; in each of these experiments a number of subjects were randomly chosen and assigned to a TST data set. Data from the remaining subjects (TRN) were used to fit models, and prediction accuracy was assessed by correlating, within-cluster, predictions and “phenotypes” in the TST data sets. In the pig data set, the testing set had a sample size of 300 for each of the clusters; therefore, in each TRN-TST partition, there were 2633 records (phenotypes and genotypes) for TRN and 900 (300 per cluster) for TST. The wheat data set is considerably smaller ($n=599$); therefore, here we only used 150 lines (86 in cluster 1 and 64 in cluster 2) for TST and 449 for TRN. As stated prediction accuracy was measured using Pearson’s product-moment correlation between predictions and phenotypes within cluster. We conducted 50 TRN-TST partitions for each of the data sets; from these analyses we report the average correlation and its SD.

## Results

### Clustering

**Fig. 3:**
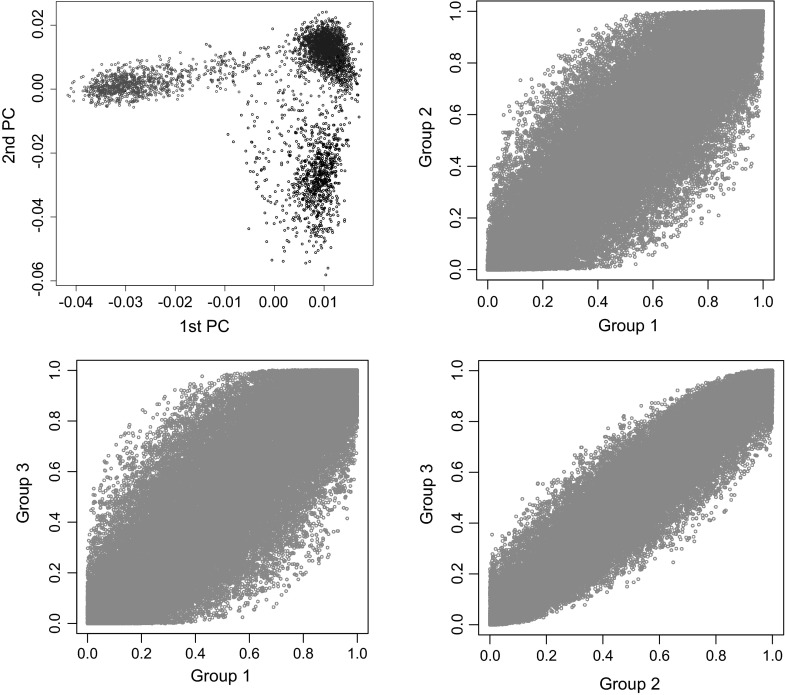
Clustering in the pig data set. First two principal components (*top-left panel*) and allele frequency (*top-right* and *lower panels*) by group (1 in *red*, 2 in *blue* and 3 in *black*).

The first two PCs and the estimated allele frequencies by cluster are presented in Fig. 1. In this data set, there were two clear clusters, one with 345 lines and the other one with 254 lines. The 1st two PCs clearly separate the two clusters inferred with PSMix, and many markers have markedly different allele frequencies in the two clusters. The results from the clustering in the pig data set are presented in Fig. 3. In this data set the 1st two PCs suggest the existence of three clusters; therefore, we implemented the clustering algorithm in PSMix with three clusters. Overall, the results from the clustering coincide with the groups that one can form by visual inspection of the first two PCs. However, for a few points, the clustering obtained with PSMix did not coincide with the classification that one could obtain based on the inspection of PCs. The degree of differentiation between clusters is clearly less marked in the pig data set than in the wheat data set: in the wheat data set, the 1st two PCs explained 16.3 % of the total variation (measured as the ratio of the sum of the two largest eigenvalues relative to the sum of all eigenvalues); this figure in the pig data set was 8.6 %, and unlike the wheat data set, allele frequencies were highly correlated between clusters in the pig data set.

### Parameter Estimates (Full-Data Analyses)

Tables 1 and 2 report estimates of variance components derived from BRR for the pig and wheat data sets, respectively. The estimated error variances of the stratified analysis were always smaller than (and in a few cases similar to) those estimated with the across-group analyses. This indicates that the stratified analyses fit the data better than a model where effects are forced to be constant across groups. On the other hand, the interaction model yielded estimates of error variance similar to those of the stratified analyses. In the pig data set, the estimated error variances were high for T3 (of the order of 0.7–0.8, indicating that the model explained between 20 and 50 % of the phenotypic variance) and lower for T4 and T5 (here the estimated error variances ranged from about 0.5 to about 0.7, depending on the trait, model, and group). On the other hand, in the wheat data set, the estimated error variances ranged from 0.491 to 0.618 for group 1, and from 0.350 to 0.657 for group 2. In group 2, the differences in estimated error variances across environments were large, with E1 (E3) showing the best (worst) model fit.

**Table 1:** Estimated posterior means of variance parameters (posterior SD) from Gaussian Model (Pig data set).

Trait	Variance	Analyses									
		Across groups			Interaction model				Stratified		
		G1	G2	G3	Main	G1	G2	G3	G1	G2	G3
T3	Genomic	0.215 (0.030)			0.064 (0.018)	0.068 (0.019)	0.051 (0.013)	0.127 (0.025)	0.309 (0.064)	0.222 (0.046)	0.335 (0.053)
	Residual	0.782 (0.052)	0.853 (0.045)	0.797 (0.035)		0.722 (0.056)	0.804 (0.046)	0.712 (0.036)	0.729 (0.057)	0.810 (0.047)	0.699 (0.037)
T4	Genomic	0.381 (0.036)			0.260 (0.042)	0.072 (0.021)	0.046 (0.011)	0.0.059 (0.016)	0.495 (0.085)	0.373 (0.060)	0.352 (0.047)
	Residual	0.602 (0.045)	0.636 (0.037)	0.675 (0.031)		0.529 (0.050)	0.604 (0.038)	0.654 (0.031)	0.553 (0.056)	0.625 (0.043)	0.673 (0.035)
T5	Genomic	0.397 (0.036)			0.280 (0.040)	0.047 (0.011)	0.053 (0.013)	0.060 (0.018)	0.419 (0.059)	0.379 (0.069)	0.349 (0.047)
	Residual	0.471 (0.036)	0.684 (0.040)	0.641 (0.029)		0.447 (0.037)	0.633 (0.043)	0.625 (0.030)	0.474 (0.040)	0.677 (0.049)	0.662 (0.034)

T3, T4, and T5 are three different traits. G1, G2, and G3 identify groups 1, 2, and 3, respectively. In the interaction model “Main” refers to the main effect and G1–G3 refer to interactions.

**Table 2:** Estimated posterior means of variance parameters (posterior SD) from Gaussian Model (wheat data set).

Environment	Variance	Analyses						
		Across groups		Interaction model			Stratified	
		G1	G2	Main	G1	G2	G1	G2
E1	Genomic	0.558 (0.095)		0.215 (0.078)	0.380 (0.121)	0.426 (0.121)	0.568 (0.133)	0.635 (0.126)
	Residual	0.605 (0.061)	0.434 (0.061)		0.554 (0.061)	0.350 (0.056)	0.563 (0.063)	0.350 (0.057)
E2	Genomic	0.497 (0.092)		0.277 (0.088)	0.308 (0.106)	0.270 (0.086)	0.600 (0.140)	0.481 (0.115)
	Residual	0.647 (0.064)	0.475 (0.062)		0.612 (0.064)	0.440 (0.062)	0.618 (0.068)	0.469 (0.067)
E3	Genomic	0.470 (0.096)	0.470 (0.096)	0.219 (0.080)	0.346 (0.115)	0.379 (0.132)	0.556 (0.135)	0.611 (0.159)
	Residual	0.531 (0.056)	0.769 (0.091)		0.490 (0.056)	0.657 (0.092)	0.491 (0.059)	0.657 (0.095)
E4	Genomic	0.485 (0.097)		0.185 (0.069)	0.378 (0.116)	0.437 (0.132)	0.568 (0.128)	0.602 (0.140)
	Residual	0.620 (0.063)	0.564 (0.076)		0.561 (0.061)	0.444 (0.070)	0.558 (0.062)	0.449 (0.071)

E1–E4 are four different mega environments. G1 and G2 identify groups 1 and 2, respectively. In the interaction model “Main” refers to the main effect and G1 and G2 refer to interactions.

In the across-group model, the genomic variance parameter is the same for all groups; whereas, in the stratified analyses, separate genomic variances are fitted for each group. Our results suggest that the estimated genomic variances from the across-group analyses were either slightly smaller or somehow in between the estimates obtained from the stratified analyses for the different groups. For instance, in the pig data set, for T3, the estimated genomic variance derived from the across-group analyses was 0.215, while the stratified analyses yielded estimates of genomic variance ranging from 0.222 (G2) to 0.335 (G3).

In the interaction model, the genomic variance can be decomposed into the main and interaction variance, and from this decomposition, one can compute the average proportion of variance explained by main effects as well as the average correlation of effects. For instance, in the pig data set (see Table 1), the percentage of variance explained by the main effect (computed from the ratio of the variance of the main effect relative to the sum of the variance of the main effects and the average interaction variance) was high (about 80 %) in T4 and T5 and intermediate to low for T3 (about 50 % for groups 1 and 2, and about 30 % for group 3). On the other hand, in the wheat data set (see Table 2), the proportion of variance explained by the main effect ranged from 30 to 50 %.

**Fig. 4:**
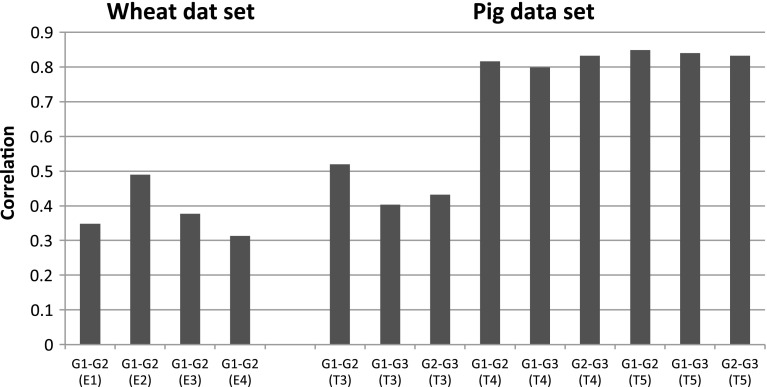
Estimated correlations between groups, by environment (wheat data set) or trait (pig data set).

In the interaction model, the average correlation of effects between groups *j* and $j'$ of any pair of groups is the ratio of the variance of the main effects divided by the square root of the product of the sum of the variances of the main and interaction effects, that is, $Cor\left( {b_{0k} +b_{jk} ,b_{0k} +b_{j{\prime }k} } \right) =\frac{Var\left( {b_{0k} } \right) }{\sqrt{Var\left( {b_{0k} +b_{jk} } \right) Var\left( {b_{0k} +b_{j{\prime }k} } \right) }}$. Figure 4 displays the estimated correlation of effects by pair of groups, traits/environments, and data sets. In the pig data set, the correlations of effects were very high (of the order of 0.8) for traits T4 and T5 and intermediate for T3 (0.4–0.5, depending on the pair of groups). Interestingly, the differences in estimated correlations were relatively large between traits and small between pairs of groups. In the wheat data set, the correlations of effects in groups 1 and 2 were considerably lower (0.3–0.5).

Tables 3 and 4 show parameter estimates derived from model BayesB for the pig and wheat data sets, respectively. The estimated error variances obtained with model BayesB were similar to those obtained with the Gaussian prior (Tables 1, 2), with no clear pattern as to whether one model fits the data better/worse than the other. The estimated proportion of markers with no-null effects (labeled “*Prob. In*” in Tables 3, 4) ranged from low (e.g., 0.269 in the stratified analyses of pig data set T3/G1) to intermediate (e.g., 0.503 in the stratified analysis of the same data set for T4/G3). In general, the estimated proportion of markers with non-null effects was smaller, and the estimated scale parameters were higher in the stratified analyses in comparison with the across-group analyses. Overall, in the stratified analyses, the proportion of markers with no-null effects was higher in the wheat data set than in the pig data set; this could be anticipated considering that marker density in the wheat data set was low and the span of linkage disequilibrium in wheat is typically long.

**Table 3:** Estimated posterior means of parameters (posterior SD) from BayesB (pig data set).

Trait	Parameter	Analyses									
		Across groups			Interaction model				Stratified		
		G1	G2	G3	Main	G1	G2	G3	G1	G2	G3
T3	Scale	2.04 (0.647)			1.09 (0.785)	0.532 (0.434)	0.624 (0.616)	0.670 (0.156)	3.760 (1.500)	1.599 (1.032)	4.421 (4.114)
	Prob. In	0.345 (0.108)			0.304 (0.154)	0.390 (0.140)	0.336 (0.188)	0.504 (0.104)	0.269 (0.099)	0.412 (0.170)	0.358 (0.196)
	Res. Var.	0.754 (0.050)	0.857 (0.045)	0.784 (0.034)		0.710 (0.057)	0.825 (0.051)	0.706 (0.035)	0.701 (0.055)	0.832 (0.049)	0.697 (0.039)
		2.504 (0.644)			2.014 (0.688)	0.455 (0.300)	0.291 (0.166)	0.182 (0.134)	5.956 (1.169)	5.891 (3.035)	2.099 (0.621)
T4	Prob. In	0.486 (0.118)			0.516 (0.144)	0.349 (0.121)	0.290 (0.115)	0.437 (0.127)	0.282 (0.064)	0.250 (0.116)	0.503 (0.117)
	Res. Var.	0.596 (0.046)	0.633 (0.037)	0.673 (0.031)		0.545 (0.051)	0.614 (0.038)	0.663 (0.031)	0.533 (0.057)	0.618 (0.043)	0.681 (0.036)
T5		2.737 (0.654)			2.556 (0.835)	0.310 (0.188)	0.352 (0.123)	0.207 (0.216)	2.983 (1.800)	2.592 (0.802)	4.500 (3.499)
	Scale Prob. In	0.426 (0.110)			0.387 (0.104)	0.343 (0.127)	0.417 (0.124)	0.384 (0.158)	0.469 (0.178)	0.445 (0.114)	0.295 (0.139)
	Res. Var.	0.460 (0.035)	0.692 (0.040)	0.632 (0.028)		0.438 (0.036)	0.634 (0.042)	0.628 (0.03)	0.450 (0.039)	0.683 (0.052)	0.659 (0.034)

T3, T4, and T5 are three different traits. G1, G2, and G3 identify groups 1, 2 and 3, respectively. Prob. In represent the estimated proportion of markers with no-null effect and Res. Var. denotes residual variance. In the interaction model Main refers to the main effect and G1–G3 refer to interactions.

**Table 4:** Estimated posterior means of parameters (posterior SD) from BayesB (wheat data set).

Environment	Parameter	Analyses						
		Across groups		Interaction model			Stratified	
		G1	G2	Main	G1	G2	G1	G2
E1	Scale	3.422 (1.231)		1.636 (1.086)	3.079 (1.884)	2.933 (1.549)	4.458 (2.471)	4.132 (1.851)
	Prob. In	0.541 (0.128)		0.463 (0.144)	0.444 (0.140)	0.481 (0.141)	0.438 (0.143)	0.510 (0.137)
	Res. Var.	0.607 (0.062)	0.451 (0.062)		0.549 (0.062)	0.364 (0.057)	0.564 (0.063)	0.368 (0.058)
E2	Scale	3.397 (1.340)		2.857 (1.427)	1.513 (1.344)	0.985 (0.770)	4.626 (2.590)	3.789 (2.268)
	Prob.-In	0.502 (0.130)		0.478 (0.136)	0.457 (0.143)	0.448 (0.142)	0.464 (0.142)	0.450 (0.144)
	Res. Var.	0.649 (0.065)	0.474 (0.062)		0.615 (0.066)	0.452 (0.064)	0.612 (0.072)	0.472 (0.070)
E3	Scale	3.287 (1.429)		2.136 (1.287)	2.255 (2.621)	2.237 (1.545)	3.932 (1.932)	4.325 (2.292)
	Prob. In	0.480 (0.135)		0.457 (0.140)	0.459 (0.143)	0.463 (0.142)	0.479 (0.137)	0.479 (0.142)
	Res. Var.	0.532 (0.056)	0.776 (0.092)		0.493 (0.058)	0.673 (0.099)	0.494 (0.061)	0.667 (0.102)
E4	Scale	3.298 (1.349)		1.322 (1.025)	2.911 (2.250)	3.267 (1.873)	3.880 (1.828)	4.268 (2.137)
	Prob.-In	0.478 (0.133)		0.457 (0.144)	0.465 (0.140)	0.469 (0.141)	0.483 (0.139)	0.480 (0.142)
	Res. Var.	0.628 (0.063)	0.576 (0.076)		0.566 (0.063)	0.456 (0.074)	0.565 (0.063)	0.463 (0.073)

E1–E4 are four different mega environments. G1 and G2 identify groups 1 and 2, respectively. Prob. In represent the estimated proportion of markers with no-null effect and Res. Var. denotes residual variance. In the interaction model Main refers to the main effect and G1 and G2 refer to interactions.

**Table 5:** Average (SD) prediction accuracy (correlation between phenotypes, average of 50 training-testing partitions), by trait, cluster and model (pig data set).

Trait	Group	BRR			BayesB		
		Across groups	Interaction model	Stratified analyses	Across groups	Interaction model	Stratified analyses
T3	1	0.213 (0.051)	0.234 (0.050)	0.231 (0.050)	0.256 (0.054)	0.257 (0.054)	0.244 (0.050)
	2	0.199 (0.039)	0.210 (0.043)	0.210 (0.044)	0.192 (0.038)	0.208 (0.042)	0.212 (0.046)
	3	0.280 (0.060)	0.301 (0.057)	0.301 (0.057)	0.297 (0.058)	0.307 (0.056)	0.304 (0.057)
T4	1	0.371 (0.042)	0.379 (0.041)	0.356 (0.041)	0.373 (0.042)	0.380 (0.041)	0.355 (0.042)
	2	0.438 (0.053)	0.424 (0.053)	0.390 (0.050)	0.439 (0.053)	0.425 (0.052)	0.391 (0.050)
	3	0.389 (0.056)	0.382 (0.053)	0.355 (0.050)	0.390 (0.055)	0.382 (0.053)	0.354 (0.050)
T5	1	0.544 (0.039)	0.541 (0.039)	0.523 (0.040)	0.564 (0.039)	0.563 (0.038)	0.550 (0.035)
	2	0.359 (0.035)	0.347 (0.036)	0.299 (0.041)	0.359 (0.034)	0.345 (0.034)	0.298 (0.041)
	3	0.401 (0.050)	0.393 (0.047)	0.368 (0.043)	0.420 (0.050)	0.410 (0.048)	0.382 (0.045)
Average		0.355	0.357	0.337	0.366	0.364	0.343

T3–T5 are three different traits.

*BRR* Bayesian Ridge Regression (Gaussian Prior).

### Prediction Accuracy

**Fig. 5:**
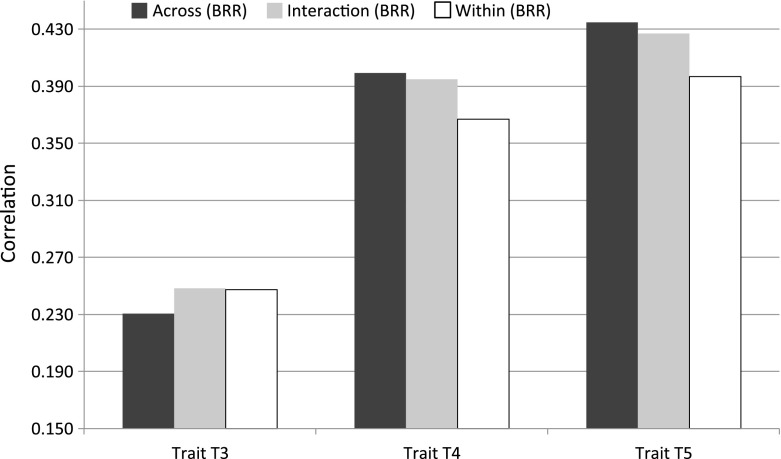
Prediction Accuracy (average over 50 training-testing partitions and 3 clusters) by trait and model, all based on model BRR (Pig data set).

Table 5 and Figure 5 show the average (SD) correlation between predictions and observations derived from 50 TRN-TST partitions for the pig data set. In this data set, the correlations between predictions and phenotypes were low (ranging from 0.2 to 0.3) for trait T3, intermediate for T4 (ranging from 0.355 to 0.439), and even slightly higher for T5 (ranging from 0.300 to 0.564). Overall, model BayesB had slightly higher prediction accuracy than the fully Gaussian model. Prediction accuracy varied between clusters; however, the patterns were not consistent across traits. Overall, there was an advantage in favor of across-group analyses relative to the stratified analyses; however, a more detailed look at the results shows that the relative rankings of these three approaches varied between traits. In T3, the stratified analyses were better than the across-group method, and the opposite happened in T4 and T5 where the advantages of the across-group model over the stratified analyses were clear. In most cases, the interaction model performed very similar to the best performing model (either the stratified or the across-group analyses), and in a few cases (e.g., in Group 1 in T3 and T4) the interaction model was the best performing one.

**Table 6:** Prediction accuracy (correlation between phenotypes, average over 50 training-testing partitions), by trait, cluster, and model (wheat data set).

Trait	Group	BRR			BayesB		
		Across groups	Interaction model	Stratified analyses	Across groups	Interaction model	Stratified analyses
E1	G1	0.453 (0.082)	0.466 (0.079)	0.455 (0.080)	0.449 (0.083)	0.467 (0.079)	0.461 (0.079)
	G2	0.573 (0.079)	0.612 (0.079)	0.613 (0.078)	0.559 (0.080)	0.604 (0.081)	0.604 (0.079)
E2	G1	0.463 (0.079)	0.463 (0.082)	0.443 (0.083)	0.453 (0.080)	0.459 (0.082)	0.440 (0.085)
	G2	0.505 (0.088)	0.488 (0.086)	0.464 (0.090)	0.501 (0.085)	0.494 (0.084)	0.462 (0.089)
E3	G1	0.410 (0.075)	0.406 (0.071)	0.396 (0.073)	0.410 (0.073)	0.408 (0.071)	0.396 (0.072)
	G2	0.362 (0.114)	0.381 (0.092)	0.373 (0.088)	0.361 (0.111)	0.376 (0.091)	0.370 (0.089)
E4	G1	0.443 (0.072)	0.460 (0.070)	0.458 (0.071)	0.442 (0.071)	0.455 (0.071)	0.454 (0.073)
	G2	0.445 (0.089)	0.487 (0.075)	0.489 (0.072)	0.442 (0.088)	0.482 (0.074)	0.481 (0.073)
Average		0.457	0.470	0.461	0.452	0.468	0.459

E1–E4 are four different mega environments.

*BRR* Bayesian Ridge Regression (Gaussian Prior).

**Fig. 6:**
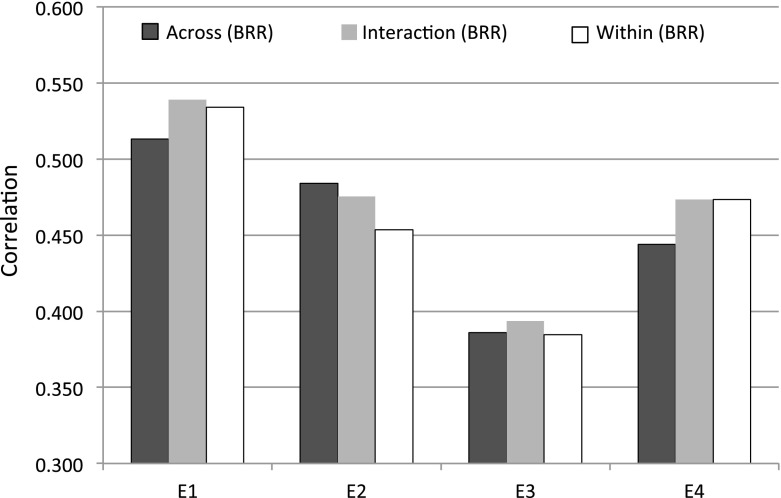
Prediction accuracy (average over 50 training-testing partitions and 3 clusters) by environment and model (all based on model BRR): wheat data set.

Table 6 and Figure 6 show the average (SD) correlation between predictions and observations derived from 50 TRN-TST partitions for the wheat data set. In this data set, prediction accuracy did not vary much between environments, and the correlations ranged from about 0.4 to about 0.6. Here, the differences between BRR and BayesB were small with only a small advantage of BRR over BayesB. In three of the four environments (E1, E2, and E4), prediction accuracy was higher in the 2nd group (G2); however, in E3, the opposite was observed. The differences between the stratified (i.e., within), across, and interaction models were also small in the wheat data set. However, in these data, we encountered three different situations. In E1 and E4, the stratified analyses were better than the across-group analyses, and the interaction model performed either similar to or sometimes better than the stratified analyses. In E2, the across-group model tended to be better than the stratified analyses, and the interaction model tended to perform either close to or better than the across-group analysis. Finally, in E3, the stratified and across-group analyses performed similarly, while the interaction model performed slightly better. Therefore, in this data set, we also observed that the interaction model was either the best performing method (see E1 and E3 in Figure 6) or tended to perform very close to the best performing method (see E4 in Fig. 6). Consequently, averaged across environments and traits, there was a slight advantage of the interaction model (see last row of Table 6).

## Discussion

Quantitative genetic theory and the models commonly used for genetic analyses of quantitative traits have been largely developed with reference to homogenous populations; concepts such as that of the average effect on an allele substitution, additive variance, and heritability are all based on the assumption of a homogenous population. However, naturally and artificially selected populations usually exhibit some degree of stratification. In the GWAS literature, stratification has been mainly dealt with as a potential source of confounding. Therefore, the focus has been on developing methods that “correct” for stratification. For example, a common practice has been to add to the regression model of interest, marker-derived PC that describes stratification as covariates (e.g., Price et al. 2010; Daetwyler et al. 2010; Yang et al. 2010; Janss et al. 2012). However, from a quantitative genetics perspective structure is more likely to act as an effect modifier than as a confounder. Indeed, differences in allele frequencies between clusters in a population are likely to induce heterogeneity of effects of allele substitution at causal loci; additionally, differences in the patterns of LD between markers and QTL may induce heterogeneity of marker effects even under the assumption that QTL effects are homogeneous.

In this article, we propose genomic regression models that incorporate structure (defined here as clusters within a population) as a potential effect modifier; we achieved this by expanding a standard whole-genome regression model with the addition of interactions between markers and cluster identifiers. The main and the interaction effects are treated as random with group-specific hyperparameters (e.g., one variance parameter for the main effects and different variance parameters for each group of interactions). This allows inferring from data the relative contribution of main and interaction effects to genomic values. In Gaussian models, this treatment also renders estimates of the average correlation of effects between sub-populations.

The statistical structure of the interaction model proposed is similar to that of models for interactions between markers and environments (e.g., the G$\times$E models described in López-Cruz et al. 2015). However, there is an important difference between interaction models for G$\times$E and methods considered here: in G$\times$E models, each subject can potentially have phenotypic records in all environments; this provides identification at the likelihood of co-dispersion parameters (variances, covariances, and correlations) even in the absence of any genetic information. On the other hand, in the models presented and used in this article, subjects belong to only one cluster; consequently, the estimation of covariance and correlations of effects between groups depends entirely on the genetic information available. For instance, in the G-BLUP models, the identification of covariance parameters (the variance of the main effects in the interaction model) depends entirely on the genomic relationships between subjects from different groups.

We evaluated the interaction model using two contrasting data sets: a wheat and a pig data set, both collected from breeding programs.

### Evidence of Heterogeneity at Genotypes

The wheat data set has two clearly defined clusters with many loci exhibiting great deal of differentiation in allele frequencies between clusters. On the other hand, the pig data set has much higher marker density than the wheat data set and has three clusters, which show some overlap, and much weaker levels of differentiation in allele frequencies than the ones observed in the wheat data set. The higher degree of differentiation of the wheat data set was also evident from the profile of eigenvalues of the genomic relationship matrix: in the wheat data set, the sum of the first 2, 5, and 10 eigenvalues was 16.3, 26.2, and 35.7 % of the sum of all eigenvalues, respectively. In the pig data set, these figures were 8.6, 11.6, and 15 %, respectively.

### Evidence of Heterogeneity of Effects

The across-group analyses yielded estimates of error variance that were systematically higher than those obtained with the stratified analyses. This suggests that forcing marker effects to be constant across sub-populations reduced the ability of the model to fit the data. On the other hand, the interaction model gave estimates of error variances that were similar to those of the stratified analyses. The variance component estimates derived from the Gaussian model suggest that the average correlation of effects is high (i.e., relatively low extent of genetic heterogeneity) in the pig data set, and much lower (i.e., much higher extent of genetic heterogeneity) in the wheat data set. This seems reasonable considering that (a) the extent of heterogeneity of allele frequencies is much higher in the wheat data set and (b) marker density in the pig data set is much higher and this is likely to make the LD patterns between markers and QTL slightly more stable across clusters. However, we also observed within the pig data set important differences in the estimated average correlation of effects across traits: for T3, the estimated correlation was considerably lower than that for T4 and T5. This illustrates an interesting feature of the model described in this article: it allows estimating trait-specific measures of genetic heterogeneity of effects between clusters in a population.

### Prediction Accuracy

We found relatively small differences in prediction accuracy due to the statistical model used, either the prior assigned to marker effects (BayesB or BRR) or whether the model was fit across clusters, within clusters, or with use of interactions. However, when averaged across traits and clusters, there was a slight superiority of the interaction model over the across-group and stratified analyses; this happened not because the interaction model was better than the other two models across traits and clusters, rather, the interaction model gave results that were more robust than the other two approaches. For instance, for some traits (e.g., T3 in the pig data set and E1 and E4 in the wheat data set) the estimated correlation of effects between clusters was low, and consequently, the stratified analysis had higher accuracy than the across-group analysis; in those cases the interaction model performed similar to the stratified analysis. On the other hand, the estimated correlation of effects in the pig data set for traits T4 and T5 was higher and in those cases the across-group model outperformed the stratified analyses and the interaction model performed close to the across-group model. A similar situation was observed for E2 in the wheat data set. Finally, for E3 in the wheat data set, the across-group and stratified analyses performed similarly and the interaction model performed slightly better.

Predictions in TST data sets are derived using information from the subjects included in the TRN data; therefore, prediction accuracy depends on how much information can be borrowed from subjects in the TRN set that is relevant to information from those in the TST set. In a G-BLUP model, borrowing of information depends on the covariances of genetic values. Within a cluster, covariances are equal to the product of the genomic relationship matrix and the genomic variance (equal to the sum of the main effect variance and the interaction variance in the interaction model). For individuals in different clusters, in the stratified analyses, the covariances are null and therefore there is no borrowing of information between clusters. On the other hand, in the across-group model, the covariance is proportional to the product of the genomic relationships and the genomic variance. However, for individuals in different clusters, the genomic relationships are usually low and consequently, there is a limited borrowing of information between individuals of different clusters, even in the across-group model. This may explain why the predictive performance of the across-group model and the stratified analyses were similar. Finally, the interaction model is somehow in between the other two approaches: in this model, the covariance of genetic values of individuals in different clusters is equal to the product of the genomic relationship and the variance of the main effect. Therefore, when the proportion of variance explained by the main effect is small (e.g., E1 and E4 in the wheat data set), the interaction model behaves similar to the stratified analyses, and when the main effect variance explains a large fraction of the total genomic variance (e.g., T4 and T5 in the pig data set), the interaction model performs similar to the across-group analyses. This may explain the relative robustness of the interaction model relative to the other two approaches.

### Advantages and Disadvantages of the Interaction Model

Several studies in the GS literature have proposed methods for analysis of clustered (generally multi-breed) data. The vast majority of the studies published so far have been based on multivariate Gaussian models (e.g., Olson et al. 2012; Karoui et al. 2012). In these models, each breed has its own set of effects and the two sets of effects are allowed to be correlated. Relative to those multivariate Gaussian models, the interaction model described in this article has advantages and disadvantages. A first advantage of the interaction model is that it can be implemented with any of the priors commonly used for genomic selection, which include not only the Gaussian prior, but also priors from the thick-tail or spike-slab families that can induce differential shrinkage of estimates and/or variable selection. Secondly, the interaction model decomposes effects in components that are common across clusters and cluster-specific deviations. This, when combined with variable selection, may lead to the identification of loci with effects that are almost constant across clusters and others whose effects are largely due to interactions. On the other hand, the interaction model also has drawbacks that are worth mentioning. Firstly, the number of effects that need to be estimated can be very large, for instance, if there are *p* markers and *q* groups, a standard interaction model requires estimating $p\times (q+1)$ effects. Secondly, since the covariance of effects is modeled via the main effects variance, the covariance is implicitly assumed to be non-negative. Moreover, whenever more than two groups are modeled, the covariance is assumed to be constant across groups. Therefore, the interaction model seems most appropriately applied in situations where the covariance of effects between groups is presumed to be positive and relatively similar between pairs of groups.

More recently, Chen et al. (2014) proposed an extension model BayesC for applications involving multi-breed data. In BayesC (Habier et al. 2011), the prior assigned to marker effects is a mixture of a point of mass at zero and a Gaussian slab. For computational convenience, these mixtures are usually implemented using two random variables: a Bernoulli random variable $(\delta _j \sim \mathrm{Bernoulli})$ that indicates the component of the mixture from where the marker effect is drawn and a normal random variable, say $b_j \sim N\left( {0,\sigma _b^2 } \right)$. Marker effects are then represented as the product of the two random variables $\beta _j =\delta _j b_j$. For multi-breed data, Chen et al. (2014) suggested to expand this representation as follows: $\beta _{1j} =\delta _j b_{1j}$ and $\beta _{2j} =\delta _j b_{2j}$, where $\beta _{1j}$ and $\beta _{2j}$ are the effects of the *j*th marker in groups 1 and 2, respectively, and $b_{1j} \sim N\left( {0,\sigma _{b1}^2 } \right)$ and $b_{2j} \sim N\left( {0,\sigma _{b2}^2 } \right)$ are treated as independent. This specification assumes that (i) that the set of markers with non-null effects (and consequently the set of segregating QTL) is the same in all groups and (ii) marker effects are uncorrelated between groups, indeed, $Cov\left( {\beta _{2j} ,\beta _{1j} } \right) =Var\left( {\delta _j } \right) Cov\left( {b_{1j} ,b_{2j} } \right) =0$. Although there are apparent similarities between the model presented by Chen et al. and the interaction model presented here, our approach does not assume that the set of segregating QTL is the same across groups, and our model also includes covariance (the variance of the main effects) between effects across groups.

### Further Extensions

Some of the limitations mentioned above can be removed by expanding the interaction model with addition of dummy variables ($-1,1$) that pre-multiply the interaction terms; these, if treated as random will allow modeling negative covariances as well. Also, whenever more than two groups are available, one can always add more interaction terms to avoid assuming that the covariance is constant across groups. However, if there are multiple groups with complex covariance patterns, the use of a multivariate Gaussian model, or the analysis based on an interaction model by pairs of groups, seems more reasonable.

In this article, we assumed that the clusters were well defined; however, admixed populations are better characterized by a continuum of variation between groups. In these cases, cluster memberships could be replaced with probabilities. For instance, if we have two groups, instead of assigning individuals to either the first or the second group, one could have the probability that a given individual belongs to group one or two. The interaction model described in this article could be extended by regarding cluster memberships as unknown and using marker-derived admixture proportions as priors to the unknown cluster memberships. Such a treatment may be useful, for instance, in the joint analyses of multi-breed and mixed-breed individuals.

Finally, at least conceptually, the interaction model presented in this article and the one considered by López-Cruz et al. (2015) can be extended to multi-environment settings by considering three-way interactions between markers, groups, and environments. For instance, if there are two groups of genotypes evaluated in two different environments, four interaction terms will be needed. Although combining interactions among three factors is conceptually possible, practical problems related to computational time and mixing of the algorithms must be considered.

## Electronic supplementary material

Supplementary material 1 (pdf 154 KB)
