# Supplementary material for: Incorporating Genetic Heterogeneity in Whole-Genome Regressions Using Interactions
Source: J Agric Biol Environ Stat. 2015 Nov 9;20(4):467–90. doi: 10.1007/s13253-015-0222-5 (PMC4666286; doi:10.1007/s13253-015-0222-5)
Supplement: Supplementary file 1 — Supplementary material 1 (pdf 154 KB) [file 13253_2015_222_MOESM1_ESM.pdf]

Supplementary Data

Table S1. Estimate Genomic Heritability derived from a Gaussian model (BRR, Pig data set).

	Analyses								
	Across groups			Interaction model			Stratified		
Trait	G1	G2	G3	G1	G2	G3	G1	G2	G3
T3	.216 (.030)	.201 (.026)	.212 (.027)	.155 (.032)	.125 (.022)	.211 (.028)	.297 (.054)	.215 (.040)	.323 (.042)
T4	.388 (.033)	.375 (.028)	.361 (.027)	.386 (.043)	.336 (.035)	.327 (.032)	.471 (.061)	.373 (.047)	.343 (.037)
T5	.458 (.034)	.367 (.028)	.382 (.027)	.422 (.041)	.345 (.036)	.352 (.031)	.468 (.049)	.358 (.053)	.344 (.038)

G1, G2 and G3 identify groups 1, 2 and 3, respectively. Main and Int. refer to main effects and interaction effects, respectively.

Table S2. Estimate Genomic Heritability derived from a Gaussian model (BRR, Wheat data set).

	Analyses					
	Across groups		Interaction model		Stratified	
Trait	G1	G2	G1	G2	G1	G2
T3	.478 (.054)	.560 (.062)	.514 (.067)	.643 (.067)	.497 (.074)	.640 (.071)
T4	.432 (.057)	.509 (.064)	.486 (.062)	.551 (.069)	.488 (.074)	.502 (.082)
T5	.466 (.065)	.378 (.062)	.531 (.069)	.473 (.078)	.525 (.079)	.477 (.087)
T4	.436 (.061)	.460 (.069)	.497 (.066)	.579 (.078)	.499 (.072)	.567 (.083)

G1 and G2 identify groups 1 and 2, respectively. Main and Int. refer to main effects and interaction effects, respectively.

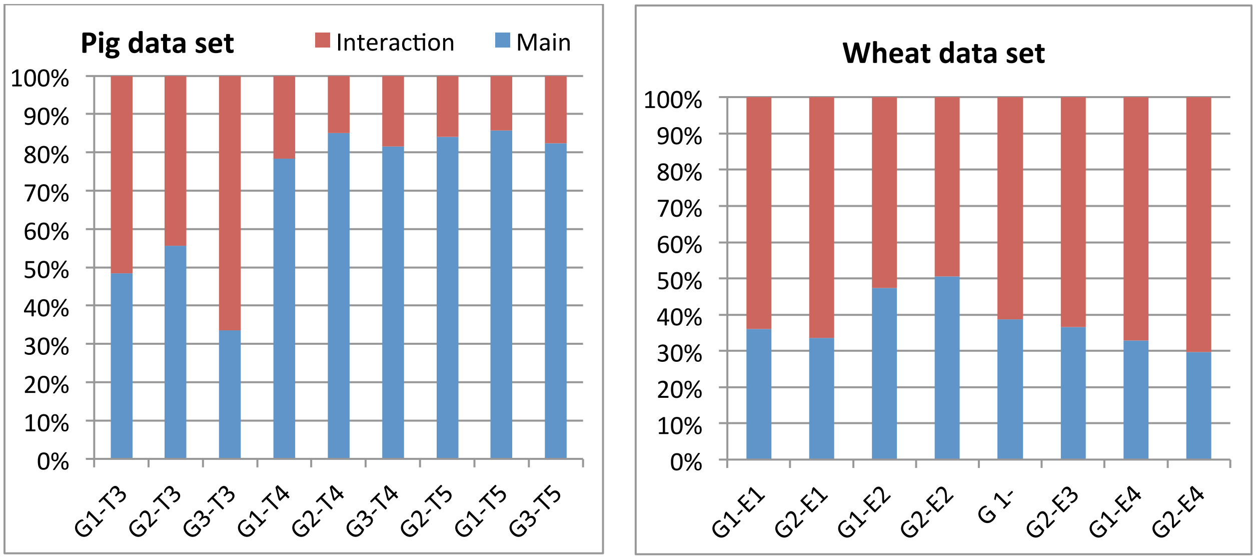

Figure 4. Proportion of the estimated genomic variance explained by the main and interaction terms by data set (pig data set in left panel, wheat data set in right panel), by group (G1-G3) and trait (T3-T5 in the pig data set)/environment (E1-E4, in the wheat data set).
